# Cardiac Surgery Training in Brazil - What Are We Discussing in Our Journal?

**DOI:** 10.21470/1678-9741-2022-0330

**Published:** 2023

**Authors:** Adnaldo da Silveira Maia

**Affiliations:** 1 Department of Cardiovascular Surgery, Instituto Dante Pazzanese de Cardiologia (IDPC), São Paulo, São Paulo, Brazil.

**Keywords:** Cardiovascular Surgery, Internship and Residency, Mentoring, Minimally Invasive Surgical Procedures, Patients, Research, Review

## Abstract

**Introduction:**

Cardiovascular surgery has undergone numerous changes over the last decades. Transcatheter technologies, endovascular procedures, hybrids, and minimally invasive surgery have undoubtedly advanced as a therapy for patients. Thus, the discussion about the training of residents in the face of new technologies in the specialty is in check. In this article, it is proposed a review to discuss the challenges in this scenario as well as the current training in cardiovascular surgery in Brazil.

**Methods:**

A comprehensive review was performed in the Brazilian Journal of Cardiovascular Surgery. All editions from 1986 to 2022 were included. The research was carried out using the search engine on the journal’s website (https://www.bjcvs.org) and an individual analysis of the titles and abstracts of each article published.

**Results:**

All the studies are summarized in the appropriate table with a discussion along this review.

**Conclusion:**

Most articles that discuss training in cardiovascular surgery in the national context are editorials and expert points of view with no observational studies evaluating the residency programs.

## INTRODUCTION

The history of cardiac surgery is characterized by great challenges. In fact, the past five decades have outlined the specialty in the way we currently conceive it. The Brazilian contribution is immeasurable, and names like Hugo Felipozzi, Euryclides de Jesus Zerbini, Adib Domingos Jatene, and Domingo Marcolino Braile, among others, are at the foundation of cardiovascular surgery^[1]^.

Cardiac surgery has been considered the culmination of surgical procedures 40 years ago. The history was changed through the work of Andreas Grüntzig in collaboration with the engineer Heinrich Hopf, who created a balloon catheter that allowed the dilatation of coronary artery stenosis in 1978. The concept of “transluminal angioplasty” had been created. Such ideas were widely criticized by the American surgical community^[2]^.

The procedure devised by Grüntzig evolved with the creation of stents, small metallic structures that would be introduced in the coronary arteries to avoid restenosis, and other techniques, such as the transcatheter aortic valve implantation (TAVI) proposed by Alan Cribier in mid-2002, that are currently performed worldwide^[2]^.

Recently, the valve-in-valve approach has replaced numerous reoperations in the percutaneous correction of dysfunction of biological valve prostheses. Such technologies can be called disruptive, as they are innovative, establishing new standards and models to the so-called traditional ones^[2,3]^.

Thus, new challenges are imposed on the training of the contemporary cardiovascular surgeon. Currently, some specialists already perform minimally invasive and endovascular procedures, which are even mandatory in some services for such a professional^[2]^. The introduction of these technologies and new approaches corroborated the change of the cardiovascular surgery residency program in 2018.

In this article, we propose a review of the Brazilian Journal of Cardiovascular Surgery about changes in medical training/residency in cardiovascular surgery as well as the medical education process involved in choosing the specialty and its ramifications.

## METHODS

This is a comprehensive review carried out in the Brazilian Journal of Cardiovascular Surgery. All editions from 1986 to 2022 were included as well as articles in Portuguese and English languages. The research was carried out in two ways:

- Using the search engine on the journal’s website (https://www.bjcvs.org) with the terms: residency OR medical residency OR training OR medical education OR cardiovascular surgery OR cardiothoracic surgery.
- Analysis of the titles and abstracts of each article published from 1986 to 2022.

We excluded articles that did not address the topic relevant to the present discussion after the search carried out by the abovementioned means.

After the search, 18 articles were included in this review (summarized in Table 1).

**Table 1:** Baseline characteristics of included studies.

Authors	Design	Year	Discussion
Andrade, JC	Experimental	1994	Teaching methodology of surgical techniques
Salerno, TA	Editorial	2002	Point of view/Experience in the specialty
Braile, D	Editorial	2006	Debate on the specialty/Importance of the Brazilian Journal of Cardiovascular Surgery and the Brazilian congress
Barbosa, GV	Editorial	2006	The need for a new residency program in cardiovascular surgery
Saadi, EK	Editorial	2007	Training in endovascular surgery
Barbosa, GV; Gomes, WJ	Editorial	2009	Training in endovascular surgery
Gomes, WJ et al.	Editorial	2009	The cardiovascular surgeon facing the emergencies of interventional procedures
Almeida, RMS	Editorial	2009	The cardiovascular surgeon and his/her relationship with interventional procedures
Braile, D	Editorial	2010	Continuing medical education
Fernandes et al.	Observational	2010	Academic leagues
Dallan, LA	Point of view	2013	Guidance on the first cases of coronary artery bypass grafting for new surgeons
Almeida et al.	Official document of the Brazilian Society of Cardiovascular Surgery	2013	Internal regulations for obtaining the title of specialist in cardiovascular surgery
Maluf et al.	Experimental	2015	Simulators for training in cardiovascular surgery
Llalle at al.	Letter to the Editor	2020	Impact of coronavirus disease 2019 on resident training
Dallan et al.	Editorial	2020	Guidelines for new surgeons
Petroianu, A	Editorial	2022	Research and cardiovascular surgery
Ribeiro TS, et al.	Point of view	2021	Medical education and its introduction to cardiovascular surgery
Barbosa, G	Editorial	2022	The new residency program in cardiovascular surgery

## RESULTS

### Training

Between the 1970s and 1990s, the Brazilian cardiovascular surgery residency program consisted in four years of training; the first year of this training was necessarily in general surgery. From the 1990s, inspired by the American program, the residency started to be carried out in four years with a two-year prerequisite in general surgery (traditional pathway). In 2006, the Sociedade Brasileira de Cirurgia Cardiovascular (SBCCV) - through resolution no 2, on September 17, 2006, from the Brazilian Ministry of Education (MEC) - created the specialization course in cardiovascular surgery (integrated pathway), dividing the specialty into two pathways:

- Traditional - with the need for a prerequisite in general surgery, lasting two years. During this period, the doctor in training is exposed to different areas, including general gastrointestinal surgery, urology, vascular surgery, and trauma surgery. After completion of general surgery training, the aspiring cardiovascular surgeons must apply for a program that varies between the institutions that offer training programs for the specialty. The Instituto Dante Pazzanese de Cardiologia offers four positions annually for its four-year cardiovascular surgery residency program.
- Integrated - this program is founded by each institution, however, there is no need for the prerequisite in general surgery; this training program lasts five years. The selection process is similar to that of the traditional pathway^[3]^.

**Fig. 1:**
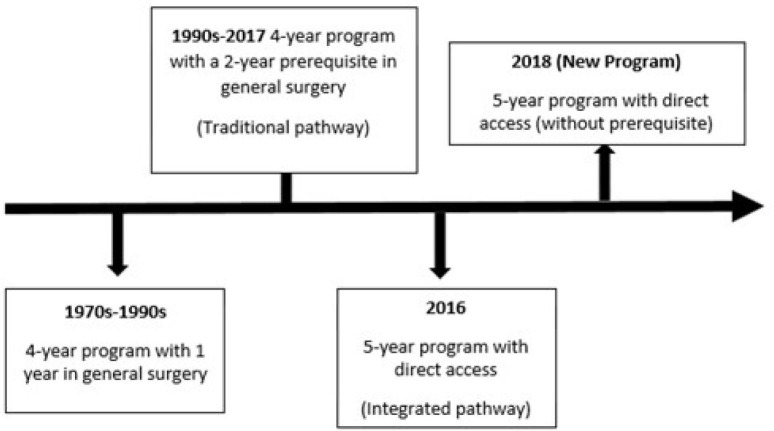
Timeline showing the evolution of medical residency programs in cardiovascular surgery in Brazil (elaborated by the author).

Both programs were regulated by the SBCCV and MEC. However, to be able to assist any patient in either the public or private health system, the cardiovascular surgeon, after completing the training in the integrated pathway, should apply for specialist title exam, offered annually by the SBCCV^[4]^ (Figure 1).

To unify the forms of training throughout the country, in 2018, after a decade of discussions involving the SBCCV and MEC, the integrated pathway was established as the only way to get into the residency program in cardiovascular surgery in Brazil. Improvements in the program were implemented to allow the resident greater contact with diagnostic methods, interventional cardiology, and endovascular surgery^[5]^.

The long training time required by the specialty was directly reflected in the historic decline in the demand for cardiovascular surgery. This debate corroborated the discussion for the change in the admission program implemented in 2018. After its implementation, large institutions registered an increase in search for positions. As an example, the Universidade de São Paulo (USP), a traditional training center, registered an increase from 3.6 applicants for each place in 2018 to 13.4 applicants for each place in 2019^[6]^. In the same period, the Instituto Dante Pazzanese de Cardiologia registered 20.6 applicants for each place, reinforcing the increase in demand for medical graduates by specialty after the change of the residency program in 2018.

In this context of increasing demand for the specialty, academic leagues play a key role. Fernandes et al.^[7]^, in an analysis of students participating in the USP cardiothoracic surgery league, showed that among final year students and recent graduates, > 50% opted for the surgical career, as well as a significant impact on the scientific production of those involved in the academic league.

The discussion of changing the medical residency program is not new. Barbosa^[8]^, in an editorial from 2006, highlights the relevance of the emergence of new technologies in the cardiovascular area and the need for the resident physician to acquire new skills, following what occurred in other specialties such as radiology, neurosurgery, and vascular surgery.

It is also observed that at that time, the discussion about the adoption of new interventional therapies was present in international congresses and highlighted by the author that such approaches would occupy part of the classically adopted procedures, especially valve surgeries^[8]^.

Such reflections proved to be true, when observing the increase in the number of procedures such as TAVI and valve-in-valve.

In this context, the processes of training and continuous learning are important for the surgeon^[9]^. Endovascular surgery is part of the cardiovascular surgeon’s field of activity, where the exchange with other specialties, such as interventional cardiology, maintenance of training centers, and consolidated programs, are factors linked to the quality of training^[10]^. In addition, Saadi^[11]^ emphasizes the importance of young surgeons in starting their practice under supervision and a direct relationship with the patient.

Such observations were also made by Almeida^[12]^, given the evolution of cardiovascular surgery and the impact on the training of the resident. The need for structure such as hemodynamics laboratories, simulators (synthetic, animal, or human cadavers or virtual reality), hybrid rooms, surgical volume, and costs are part of the training challenges.

The proposal of simulators and new teaching techniques applied to medical residency are described in the literature. Andrade^[13]^ proposed the implementation of a surgical room with mannequins and anatomical parts to perform different surgical techniques, including aortic annulus enlargement, Jatene surgery, heart transplantation, and mitral valve repair. Similarly, Maluf et al.^[14]^ demonstrated the applicability of a simulator for training coronary anastomoses, contributing to the development of such skills for surgeons in training.

### Challenges

The field of cardiovascular surgery is complex and continues to evolve. The last few decades have brought enormous changes to the practice of cardiovascular surgeons. For example, the approach to the aortic valve through transcatheter techniques, image-guided procedures, and minithoracotomy are currently treatment modalities that are part of the surgeon’s arsenal^[15]^. So, how to implement such changes in the residency training? How to keep the vanguard as a specialty?

The future of cardiovascular surgery has been extensively discussed by numerous authors. Braile^[16]^ and Salerno^[17]^ highlight some points such as the teaching offered by institutions linked to graduate studies, the incorporation of new less invasive procedures, and the need for cardiovascular surgeons to be able to take new approaches in order to respond to the desires of the new generation of surgeons.

It is interesting to note that the possibility of performing endovascular procedures or implantation of devices by other medical specialties are relegated to cardiovascular surgeons, what Gomes et al.^[18]^ described as “rescue surgeons”. This denomination refers to the surgeons who would act in possible complications secondary to these procedures. This reflection again allows us to sustain the importance of incorporating these procedures into the surgeon’s daily practice.

Dallan^[19,20]^, in this challenging context of the specialty, makes a historical review of cardiovascular surgery. Its evolution throughout the 20^th^ century, with the performance of the first surgeries and development of extracorporeal circulation, reinforces the Brazilian relevance with distinguished surgeons such as Euryclides de Jesus Zerbini and Adib Domingos Jatene. The future of the specialty passes, according to the author, through the realization of the increase in population life expectancy and the diseases inherent to this fact, the increase in use of virtual reality, and the possibility of performing long-distance surgery. However, such aspects would be secondary in view of the need for a leader in the face of the procedures.

Petroainu^[21]^, in a recent editorial in the Brazilian Journal of Cardiovascular Surgery, describes the importance of research in the training of cardiovascular surgeons. The restricted Brazilian funding - in general, from the Conselho Nacional de Desenvolvimento Científico e Tecnológico (or CNPq), Coordenação de Aperfeiçoamento de Pessoal de Nível Superior (or CAPES), or Fundações de Apoio à Pesquisa (or FAPs) - limits scientific production. In addition to this fact, the impact of the coronavirus disease 2019 pandemic on the economic and social scenarios, not only nationally, has been highlighted in recent years, promoting indirect and direct effects on the training of residents^[22]^.

At the invitation of the Associação Brasileira dos Residentes de Cirurgia Cardiovascular (or ABRECCV), Barbosa^[23]^, one of those responsible for engaging the change in the medical residency program in cardiovascular surgery, proposes some reflections, including the idea that the pedagogical project of teaching and training of the new program aims to produce a new cardiovascular surgeon with new skills. Integration with other areas of cardiology, hemodynamics, vascular, and thoracic surgery is in line with this concept.

In addition, the author highlights some challenges for new surgeons:

- New paradigms need to be understood and accepted.
- Adaptation to new procedures and technologies is necessary.
- Actively participate in the Heart Team, not just as a secondary professional.
- New surgeons need to maintain the habit of continuous learning.

### Limitations

There were few limitations in this review such as a small number of studies included and a single source of them (the Brazilian Journal of Cardiovascular Surgery). Although in the absence of a large study discussing the cardiovascular surgery training in Brazil, this review could provide some reference about.

## CONCLUSION

Most articles that discuss training in cardiovascular surgery in the national context are editorials and expert points of view. Thus, there are no observational studies that evaluate residency programs, similar to what has been shown in other countries. It is necessary to keep this discussion present in order to promote the continuous improvement of the training of Brazilian cardiovascular surgeons.

## References

[1] Braile DM, Godoy MF (2012). History of heart surgery in the world. 1996. Rev Bras Cir Cardiovasc.

[2] Braile DM, Zanini M, Gonçalves CSA, Evora PRB (2019). Heart surgery and disruptive technology. Braz J Cardiovasc Surg.

[3] Rocha RV, Almeida RMS (2018). Cardiac surgery residency in Brazil: how to deal with the
challenges of this unique specialty. J Thorac Cardiovasc Surg.

[4] Almeida RM, Leal JC, Murad H, Jatene F, Braile D (2013). Regimento interno para a obtenção do título
de especialista - sociedade brasileira de cirurgia
cardiovascular. Rev Bras Cir Cardiovasc.

[5] Dunning J (2019). Disruptive technology will transform what we think of as robotic
surgery in under ten years. Ann Cardiothorac Surg.

[6] Ribeiro TS, Faria RM, Santos MAD (2021). Medical school, cardiovascular surgery, and education: how we do
it in Brazil's scenario?. Braz J Cardiovasc Surg.

[7] Fernandes FG, Hortêncio Lde O, Unterpertinger Fdo V, Waisberg DR, Pêgo-Fernandes PM, Jatene FB (2010). Cardiothoracic surgery league from university of São Paulo
medical school: twelve years in medical education experience. Rev Bras Cir Cardiovasc.

[8] Barbosa GV (2006). A new medical residence program in cardiovascular surgery with
direct access. Braz J Cardiovasc Surg.

[9] Braile DM (2010). Continuing Medical Education: essential. Braz J Cardiovasc Surg.

[10] Barbosa GV, Gomes WJ (2009). A sociedade brasileira de cirurgia cardiovascular e a
inserção dos cirurgiões cardiovasculares como
especialistas endovasculares. Rev Bras Cir Cardiovasc.

[11] Saadi EK (2007). Is it possible to train an endovascular surgeon?. Rev Bras Cir Cardiovasc.

[12] Almeida RM (2009). O cirurgião cardiovascular como
intervencionista. Rev Bras Cir Cardiovasc.

[13] Andrade JC (1994). Nova metodologia para ensino e ensaio de técnicas
operatórias em cirurgia cardíaca. Rev Bras Cir Cardiovasc.

[14] Maluf MA, Gomes WJ, Bras AM, Araújo TC, Mota AL, Cardoso CC (2015). Cardiovascular surgery residency program: training coronary
anastomosis using the arroyo simulator and UNIFESP models. Braz J Cardiovasc Surg.

[15] Pelletier MP, Kaneko T, Peterson MD, Thourani VH (2017). From sutures to wires: the evolving necessities of cardiac
surgery training. J Thorac Cardiovasc Surg.

[16] Braile D (2006). The future of cardiovascular surgery. Braz J Cardiovasc Surg.

[17] Salerno TA (2002). A realistic view of the cardiothoracic surgery
specialty. Rev Bras Cir Cardiovasc.

[18] Gomes WJ, Almeida RM, Braile D (2009). O cirurgião de resgate. Braz J Cardiovasc Surg.

[19] Dallan LAO, Jatene FB (2020). Perspectives of the young cardiovascular surgeon. Braz J Cardiovasc Surg.

[20] Dallan LA (2013). Words to the young cardiovascular surgeon. Rev Bras Cir Cardiovasc.

[21] Petroianu A (2022). Research in the training of cardiovascular
surgeon. Braz J Cardiovasc Surg.

[22] Llalle WSC, Bellido-Yarlequé D, Yépez-Calderón C, Chávarry-Infante P (2020). Impact on the thoracic and cardiovascular surgery residents'
learning curve during the COVID-19 pandemic. Braz J Cardiovasc Surg.

[23] Barbosa GV (2022). Change in the SBCCV's medical residency program - perspectives of
the specialty and challenges of young surgeons. Braz J Cardiovasc Surg.

